# Chemical Records in Snowpits from High Altitude Glaciers in the Tibetan Plateau and Its Surroundings

**DOI:** 10.1371/journal.pone.0155232

**Published:** 2016-05-17

**Authors:** Yulan Zhang, Shichang Kang, Qianggong Zhang, Tanguang Gao, Junming Guo, Bjorn Grigholm, Jie Huang, Mika Sillanpää, Xiaofei Li, Wentao Du, Yang Li, Xinlei Ge

**Affiliations:** 1 State Key Laboratory of Cryospheric Sciences, Cold and Arid Regions Environmental and Engineering Research Institute, Chinese Academy of Sciences, Lanzhou, China; 2 Laboratory of Green Chemistry, Lappeenranta University of Technology, Sammonkatu 12, FI-Mikkeli, Finland; 3 CAS Center for Excellence in Tibetan Plateau Earth Sciences, Chinese Academy of Sciences, Beijing, China; 4 Key Laboratory of Tibetan Environment Changes and Land Surface Processes, Institute of Tibetan Plateau Research, Chinese Academy of Sciences, Beijing, China; 5 Key laboratory of Western China’s Environmental System (Ministry of Education), College of Earth and Environmental Sciences, Lanzhou University, Lanzhou, China; 6 Climate Change Institute and Department of Earth Sciences, University of Maine, Orono, Maine, United States of America; 7 Jiangsu Key Laboratory of Atmospheric Environment Monitoring and Pollution Control (AEMPC), School of Environmental Sciences and Engineering, Nanjing University of Information Science and Technology, Nanjing, China; Institute of Tibetan Plateau Research, CHINA

## Abstract

Glaciochemistry can provide important information about climatic change and environmental conditions, as well as for testing regional and global atmospheric trace transport models. In this study, δ^18^O and selected chemical constituents records in snowpits collected from eight glaciers in the Tibetan Plateau and adjacent areas have been investigated. Drawing on the integrated data, our study summarized the seasonal and spatial characteristics of snow chemistry, and their potential sources. Distinct seasonal patterns of δ^18^O values in snowpits indicated more negative in the south TP controlled by Indian monsoon, and less negative in the north TP and Tien Shan. Overall increasing concentrations of microparticles and crustal ions from south to north indicated a strength of dust deposition on glaciers from semi-arid and arid regions. Principal component analysis and air mass trajectories suggested that chemical constituents were mainly attributable to crustal sources as demonstrated by the high concentrations of ions occurring during the non-monsoon seasons. Nevertheless, other sources, such as anthropogenic pollution, played an important role on chemical variations of glaciers near the human activity centers. This study concluded that air mass transport from different sources played important roles on the spatial distributions and seasonality of glaciochemistry.

## 1. Introduction

Glaciers are depositional sites of atmospheric chemical components and a fascinating laboratory for the study of atmospheric chemistry. Glaciochemistry can provide important information on climatic change [1–3], environmental conditions [4–8], and data for testing regional and global climate-chemistry models [9–10]).

The Tibetan Plateau (TP) and its surroundings are referred to as the “Third Pole”, containing the largest number of glaciers outside the Polar Regions [11]. Due to its high altitude and large area, TP plays a key role in climatology of Asia [12], with its unique and complex interactions of atmospheric, cryospheric, hydrological, geological and environmental processes bearing a large effect on the Earth’s biodiversity, climate and water cycles [11]. Because of its relatively sparse human activities, glaciochemistry records retrieved from the TP provide us an opportunity to monitor past and present climate and environmental changes [1, 3, 7, 13–14], and evaluate how human activities have impacted on climate and environment [1, 14].

Based on the glaciochemical data set (Ca^2+^, Na^+^, Cl^-^, SO_4_^2-^, NO_3_^-^), Wake et al. [5] pointed out that the TP was dominated by desert dust derived from the vast arid and semi-arid regions of central Asia, while snow from the southern slope of Himalaya was characterized by very low ion burdens. On average, the flux of major ion in the central TP (Mt. Tanggula) is 6–30 time higher than that of the region to the south, and 0.6–5 times higher than regions to the southeast [15]. These studies provide an understanding of the glacialchemical pattern in outline in almost twenty years ago. Glacial chemistry has also been studied to understand the seasonality of atmospheric deposition, for example, in the Mt. Everest region [4], Tanggula Mts. [16], Muztagata [13, 17], Qilian Mts. [18], and Tien Shan [19], which are mainly focused on the individual glacier. How is the present glacial chemistry under the climate change and its relationship with air mass transportation in the different region of the TP? Widespread glaciers across the TP offer us an opportunity to understand the environmental evolution of the plateau.

**Fig 1:**
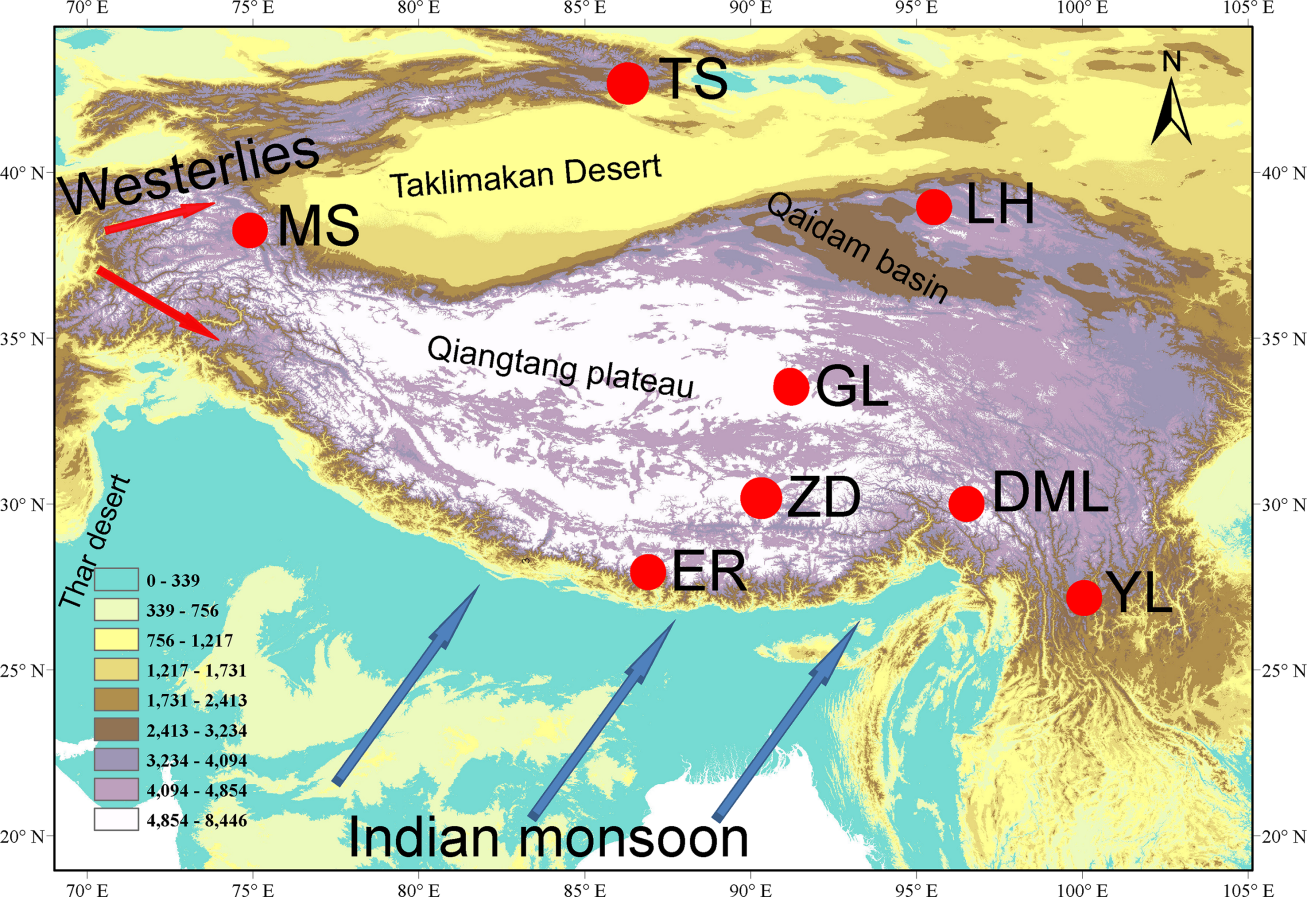
Location map showing the snowpit sites on the Tibetan Plateau and adjacent areas. (TS: Ürümqi Glacier No. 1, Tien Shan; LH: Laohugou Glacier No.12, Mt. Qilian; MS: Muztagata Glacier; GL: Guoqu Glacier, Mt. Geladiandong; ZD: Zhadang Glacier, Mt. Nyainqengtanglha; ER: East Rongbuk Glacier, Mt. Everest; DML: Demula Glacier; and YL: Yulong Snow Mountain. Detailed information is given in Table 1).

In this study, we will focus on δ^18^O and selected chemical compounds in snowpits collected from eight glaciers from the southern to the northern TP and adjacent areas (Fig 1), and discuss the impact of air mass transport on seasonal chemical records. This study summarizes the seasonal and spatial characteristics of glaciochemistry and the influence of long-range transport air masses on the measured chemical concentrations in the different regions. In an attempt to evaluate the potential sources of glaciochemistry, we compare the spatial distributions of chemicals in the snowpits with data from the National Centers for Environmental Prediction/National Center for Atmospheric Research (NCEP/NCAR) vector wind field. The data we report in this paper will provide further insight into the spatial and temporal distributions of major chemicals deposited on glaciers across the TP, which may be useful for the simulation of aerosol on the global/regional climate effect.

## 2. Methodology

### 2.1 Study area and snow sample collecting

This study was carried out in the Qinghai-Tibet (Tibetan) Plateau and Xinjiang Autonomous Region with no specific permissions required for Chinese scientists. Field work procedure was in strict accordance with the environmental protection required by the Cold and Arid Regions Environmental and Engineering Research Institute and the Institute of Tibetan Plateau Research, Chinese Academy of Sciences. Field sampling sites in our study did not involve endangered or protected species. The results will benefit local people and attract public attentions to the cryospheric environmental conditions.

The TP and its adjacent areas, referred as the “Third Pole” and the “Asian Water Tower,” cover an area of 5 million km^2^ with an average altitude of more than 4000 m a.s.l. and contain the largest number of glaciers outside the Polar Regions [11]. As a result of rising temperatures since the mid-1950s, most glaciers in the TP are experiencing shrinkage [20], impacting water availability and the environment [8, 21].

The TP stretches from the Pamir and Hindu Kush in the west to the Hengduan Mountains in the east, and from the Himalayas in the south to the Kunlun and Qilian Mountains in the north [20]. Due to its high altitude and varying topography, the TP exerts profound thermal and dynamic influences on atmospheric circulation [12] and divides the westerlies, forcing them to flow around it in winter. It also acts as an important source of potential vorticity for summer atmospheric movement related to the Indian monsoon. Both the westerlies and the Indian monsoon profoundly affect the advection heat, moisture transport, and climate patterns in the TP region [12].

During 2008–2010, we collected samples from eight snowpits to investigate the glaciochemistry of high altitude glaciers in the TP (Fig 1 and Table 1). Detailed information of the sampling sites from Ürümqi glacier No.1 (TS, Eastern Tienshan), Laohugou Glacier No.12 (LH, Qianlian Mountain), Muztagata glacier (MS, the eastern Pamir), Guoqu glacier (GL, Mt. Geladaindong), Zhadang glacier (ZD, Mt. Nyainqêngtanglha), East Rongbuk glacier (ER, Mt. Everst), Demula glacier (DML, southeast Tibetan Plateau) and Yulong Snow Mountain (YL) are shown in S1 Text. The samples were collected at a vertical resolution of 5 cm or 10 cm using a pre-cleaned plastic scoop. Polypropylene cleanroom suits and non-powder vinyl cleanroom gloves were worn at all times to minimize potential contamination of samples. The density of the snow samples was also measured on site. The snow samples were transferred into whirl-pak bags, refrigerated at –20°C, and kept frozen during transportation. In the laboratory they were melted at room temperature and transferred into pre-cleaned containers (HDPE).

**Table 1:** Sampling information for the studied glaciers in the Tibetan Plateau and adjacent areas.

Sites	Sampling Time	Lat. (°)	Lon. (°)	Alt. (m a.s.l.)	Snowpit depth (cm)	Sample numbers	Monsoon	Non-monsoon
**Ürümqi glacier No. 1 (TS)**	2008-10-20	43.11	86.81	4063	215	22	Jul-Sep in 2007 and 2008	Oct 2007—May 2008 Oct 2008
**Laohugou glacier No.12 (LH)**	2008-10-16	39.43	96.56	5026	125	13	Jul-Sep 2008	Jan-Jun and Oct in 2008
**Muztagata glacier (MS)**	2010-7-18	38.29	75.05	5725	145	15	Jun-Jul 2010 and Jun-Sep 2009	Oct 2009-May 2010
**Guoqu glacier (GL)**	2009-4-23	33.58	91.18	5765	65	7	Jun-Sep 2008	Oct 2008- Apr23 2009
**Zhadang glacier (ZD)**	2009-5-7	30.47	90.65	5797	205	21	Jun-Sep 2008	Oct 2008- May7 2009
**East Rongbuk glacier (ER)**	2009-5-18	28.02	86.96	6525	105	11	Jun-Sep 2008	Jan-May 2009
**Demula glacier (DML)**	2008-9-21	29.36	97.02	5404	175	18	Jun-Sep 2008	Oct 2007- May 2008
**Yulong Snow mountain (YL)**	2009-5-20	27.11	100.20	4747	282.5	15	Jun-Sep 2008	Oct 2008- May20 2009

### 2.2 Experiment

#### 2.2.1 Oxygen isotope analysis

Refrigerated, well-sealed snowpit samples were analyzed for δ^18^O at the Key Laboratory of Tibetan Environment Changes and Land Surface Processes, Institute of Tibetan Plateau Research, Chinese Academy of Sciences (TELCAS, http://www.tel.itpcas.ac.cn/), using MAT-253 Isotope Mass Spectrometer (±0.2‰ precision) via the standard CO_2_ equilibration technique. All data were reported in the standard δ notation vs. the Vienna Standard Mean Ocean Water.

#### 2.2.2 Major ion analyses

Also at TELCAS, all samples were analyzed for major cations (Na^+^, K^+^, Ca^2+^, Mg^2+^, NH_4_^+^) and major anions (Cl^–^, SO_4_^2–^, NO_3_^–^) using the Dionex 2000 system with a 200-μl sample loop and the Dionex 2500 system, respectively [22]. Samples for cation analysis were eluted on Dionex CS12A 4 mm separatory column with a CS12A 4 mm guard column using 20 mM MSA (Methanesulfonic acid) solution for an eluent pumped flow rate of 1.0 mL min^–1^; suppression was provided by a Dionex CSRS suppressor in recycle mode. Samples for anion analysis was analyzed by Dionex 2500 ion chromatograph using an IonPac AsII-HC column, 25 mM KOH eluent and ASRS suppresser. The analytical detection limit was 1 ng/g for all the ions. The precision of the measurements is less than 5%.

#### 2.2.3 Microparticles

Microparticle concentrations were detected by using a 256-channel Coulter Counter in a class-100 clean room at the State Key Laboratory of Cryospheric Sciences, Arid and Cold Region Environmental and Engineering Research Institute, CAS. Since the Coulter Counter counted the microparticles by volume, the microparticle sizes used were spherical equivalent diameters larger than 1.0 μm [16].

## 3. Results

### 3.1 Profiles of δ^18^O from snowpits

**Fig 2:**
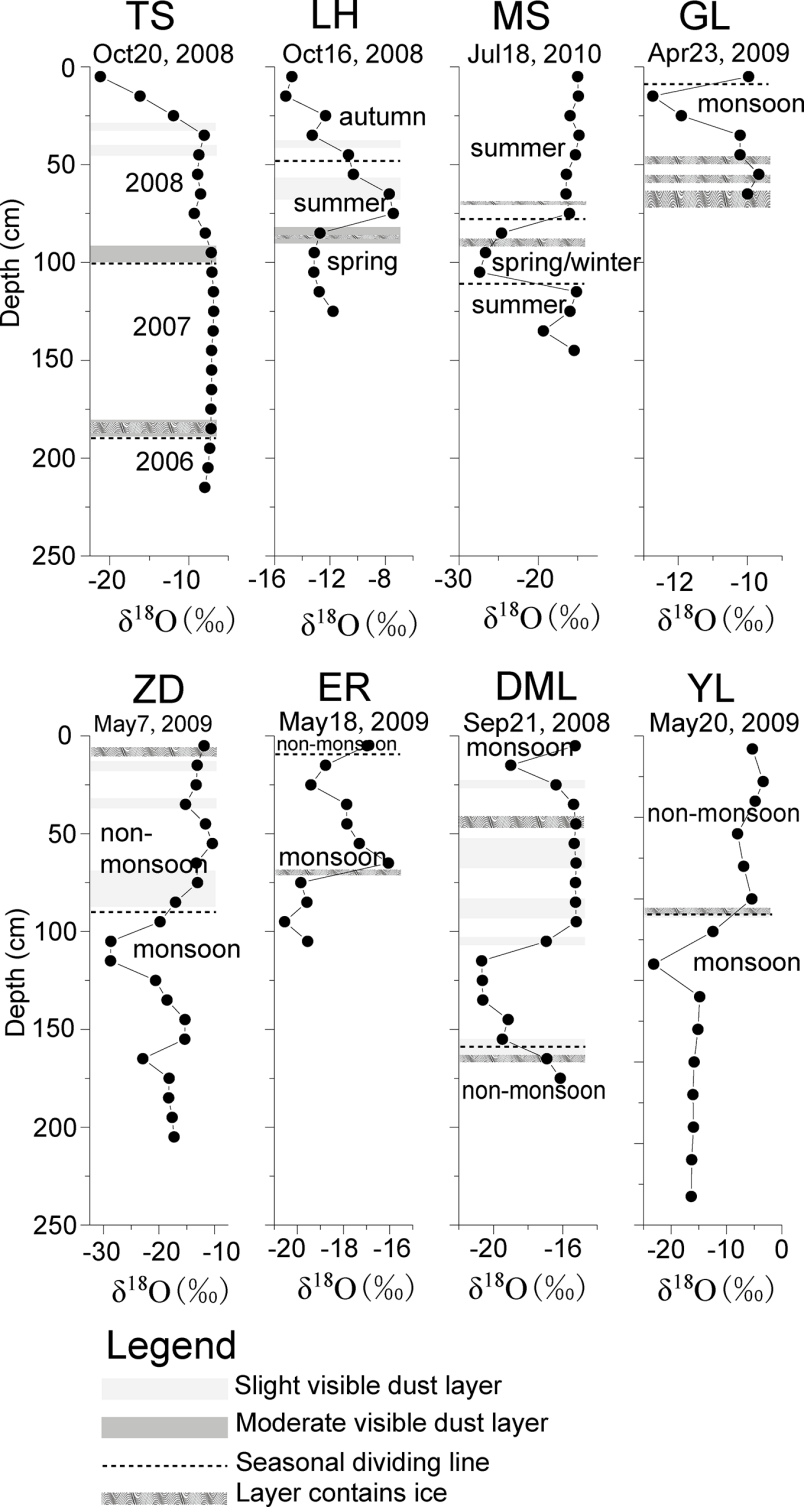
Variations of δ^18^O in the eight snowpits and their seasonal divisions. (TS: Ürümqi Glacier No. 1, Tien Shan; LH: Laohugou Glacier No.12, Mt. Qilian; MS: Muztagata Glacier; GL: Guoqu Glacier, Mt. Geladiandong; ZD: Zhadang Galcier, Mt. Nyainqêngtanglha; ER: East Rongbuk Glacier, Mt. Everest; DML: Demula Glacier; and YL: Yulong Snow Mountain. Detailed information listed in S1 Table.)

In our study, variations of δ^18^O with snowpit depth are shown in Fig 2. During the sampling, the depth of visible dust layer and ice layer appeared in the snowpits was marked. For Tienshan (TS), seasonal accumulation were determined based on the perceptible dust/ice layers because the snowpit subjected to intensive melting during summer season can form a major dust layer each year [23]. The top dust layer in TS in the depth of ~40cm was formed in the late 2008 summer (Fig 2). For Laohugou (LH) and Muztagata (MS), higher δ^18^O values correspond to higher temperature during summer time [24]. Determination of seasonality for other snowpits in the monsoon affected region were basically followed the rule of “δ^18^O is more negative in monsoon season while less negative in non-monsoon season”, combined with the consideration of regional annual precipitation amount [25–26]. For Everest (ER), most of the non-monsoon snowfall was blown away with little accumulation from observation. For Demula (DML) in the southeastern Tibetan Plateau, seasonality was determined by the ice layer formed when the strong summer melt occurred [8].

In eastern Tien Shan, which has a pronounced continental climate, the maximum of precipitation occurred in spring and early summer [2]. In recent years, the glaciers on Tien Shan have experienced an accelerated loss of ice [2] and the water from the glacial melt has influenced the δ^18^O records. Tian et al. [25] found that the δ^18^O in precipitation was coincident with air temperature, with less negative δ^18^O values in summer. In our snow samples from Glacier No. 1 in the headwater of Ürümqi river in Tien Shan, the δ^18^O values are completely smoothed. Wang et al. [27] reported that when the air temperature was high, the δ^18^O oscillations in snowpits may be rapidly altered in presence of percolating meltwater. The effects of meltwater elution on the TS glaciers clearly smoothed the isotopic signals as shown in Fig 2.

The northern and western parts of the TP are considered to be less affected by Indian monsoon. The δ^18^O records from these regions (Laohugou Glacier No.12 (LH), and Muztagata Glacier (MS)) showed a distinct seasonality with less negative values in summer and more negative values in winter, similar to previous results from the northern and western TP [13, 15, 17–18, 24–26].

In the central-southern TP, the δ^18^O values in the Guoqu Glacier on Mt. Geladiandong (GL), Zhadang Galcier on Mt. Nyainqéngtanglha (ZD), and East Rongbuk Glacier on Mt. Everest (ER) were more negative during the summer monsoon and less negative in winter as a result of the impact of Indian monsoon precipitation (Fig 2). Overall, the mean δ^18^O values are slightly depleted from the northern to southern TP (Table 1, Fig 1). In the southeastern part of the TP, the δ^18^O records from the Demula Glacier (DML) and Yulong Snow Mountain (YL) exhibited lower values during monsoon seasons (Fig 2). The various seasonality of δ^18^O records indicated that different moisture sources and different transport pathways may result in different temporal variations of δ^18^O in snowpits.

Isotopic composition of precipitation is influenced by the evaporation and condensation history of the associated air mass, and is closely linked to climatic parameters such as surface air temperature, precipitation, and relative humidity [28]. Yao et al. [26] noted that the spatial and temporal patterns of precipitation in the TP revealed three distinct domains: one associated with the influence of the westerlies (northern TP), one associated with the Indian monsoon (southern TP), and a transitional pattern between the two. Specifically, in the northern TP under the control of westerlies, variations of δ^18^O in precipitation depended on air temperature (less negative in summer, as shown by MS), while in the southern TP under the control of Indian monsoon in summer, the quantities of isotopes in precipitation appeared to be influenced more by the amount of precipitation (more negative in summer, as shown by ER). During monsoon season (June to September), low pressure over the plateau induces a pathway of moist, warm air mass from the Indian and Pacific Oceans to the TP, resulting the δ^18^O in the south TP controlled by the effect of precipitation amount [25]. While during the non-monsoon season, high pressure drives cold, dry air out of the plateau [12, 29]. Therefore, different patterns of δ^18^O values in TP snow/precipitation were related to the impact of the moisture sources in different seasons [26, 30].

### 3.2 Major ion concentrations and variations

Average concentrations of major ions measured in the snowpits are presented in Table 2. Overall, concentrations of Ca^2+^ and Mg^2+^, typical crustal constituents, gradually increased from south to north among the eight sampled glaciers, with the maximum concentrations occurring at LH, near the desert areas of Northwest China. The higher concentrations of NH_4_^+^, SO_4_^2–^, and NO_3_^–^ present at TS, LH, ZD and YL may be affected by anthropogenic pollution transportation and deposition, which is located near the city (TS, LH and YL, see the S1 Text) or facilitated with pasture activities (ZD) [22, 31–32]. The highest concentrations of Na^+^, K^+^, and Cl^–^, the sea-salt and crustal components [22, 32], appeared at ER and ZD. On average, the major ions in the snowpits were dominated by Ca^2+^ (~40%), with important contributions from SO_4_^2–^ and NO_3_^–^. However at ER, Na^+^, Cl^–^, and SO_4_^2–^ represented 18.8%, 22.6%, and 24.1%, respectively, of total ion concentrations; Ca^2+^ only represented 11.2%.

**Table 2:** Mean values of δ^18^O (‰), major ions (ng/g), and microparticles (10^3^ /mL) in studied snowpits.

Sites	δ^18^O	Na^+^	NH_4_^+^	K^+^	Mg^2+^	Ca^2+^	Cl^–^	SO_4_^2–^	NO_3_^–^	Microparticles
**TS**	–8.84	140	213	59.4	194	1159	256	593	359	894
**LH**	–11.94	149	163	30.9	345	1855	364	547	356	740
**MS**	–17.97	75.9	118	40.0	49.4	506	110	109	154	197
**GL**	–10.67	176	142	31. 8	85.0	1186	263	376	291	170
**ZD**	–17.14	352	210	146	76.1	1658	363	444	256	562
**ER**	–18.53	327	163	108	27.5	194	392	419	109	36.5
**DML**	–17.12	14.7	19.6	20.6	30.5	129	30.5	29.2	52.9	205
**YL**	–12.00	207	229	65.2	53.5	1003	245	459	220	116

TS, LH, MS, GL, ZD, ER, DML, and YL are the same as in Table 1.

**Fig 3:**
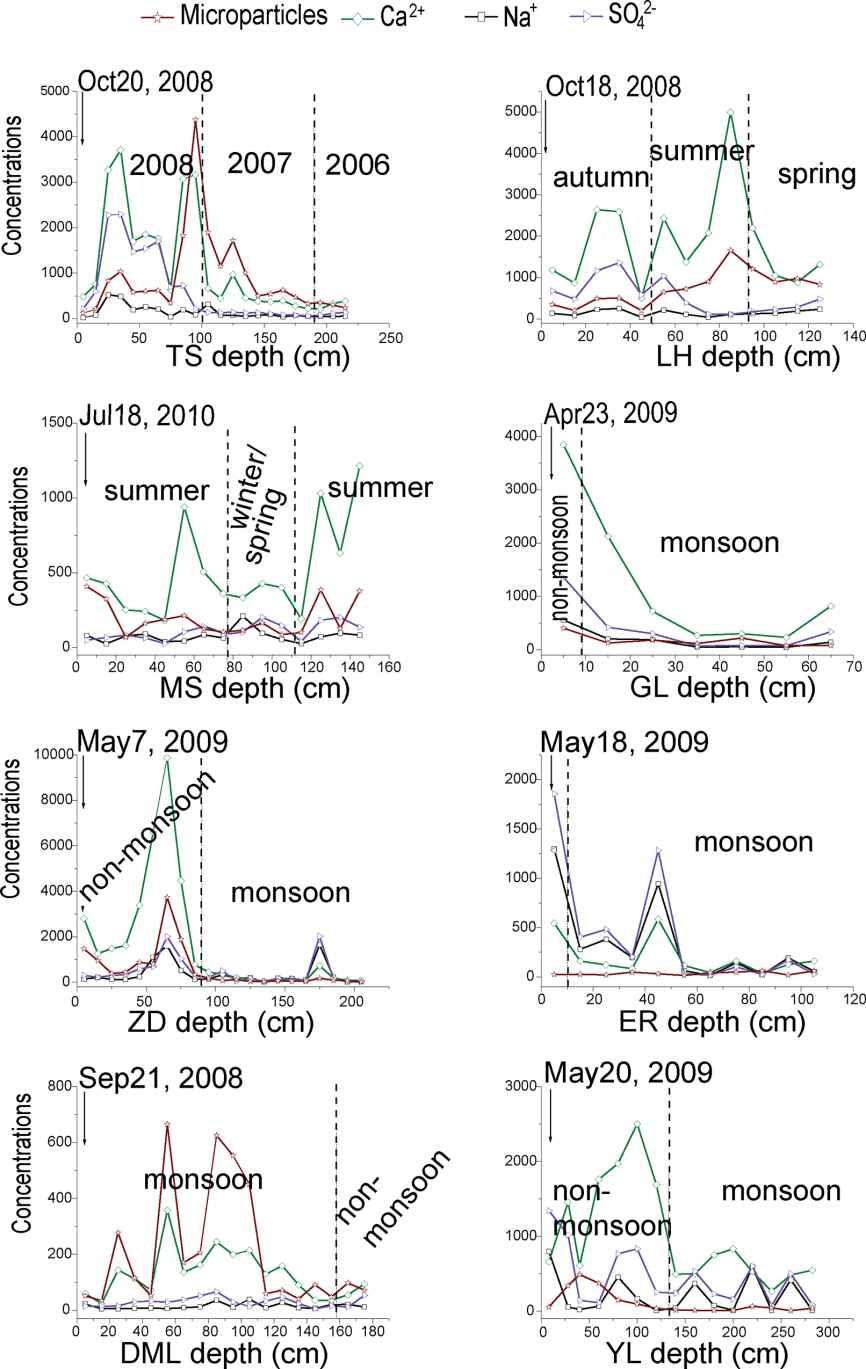
Variations of selected ions (ng/g) and microparticles (×10^3^/mL) from the eight snowpits in Tibetan Plateau and adjacent areas. (Vertical dashed line represents seasonal boundary according to Fig 2; TS, LH, MS, GL, ZD, ER, DML, and YL are the same as in Fig 2)

Variations of selected ions (Ca^2+^, Na^+^, SO_4_^2-^) and microparticles in snowpits are presented in Fig 3. At each site, the concentration curves displayed similar trends. Particularly at GL, ZD, ER, DML, and YL, major ions showed visible seasonality, with higher values in the non-monsoon seasons and lower values in the monsoon season. This is a normal phenomenon previously reported by Kang et al.[33] and Zhang et al. [16]. In contrast, at LH and MS, high values occurred in summer [26]. Microparticles and chemical concentrations possessed similar variations (Fig 3). As crustal proxies, microparticles, Ca^2+^, and Mg^2+^ displayed subtle differences. Microparticles and chemical species are removed from the atmosphere through dry and wet deposition [34]. For both types of deposition, precipitation and wind are the main control factors on glaciers. After the aerosols accumulated on the glacial surface, they undergo post-depositional alteration influenced by temperature and meltwater percolation. Thus, microparticles deposition plays an important role in the chemical composition of snow.

**Fig 4:**
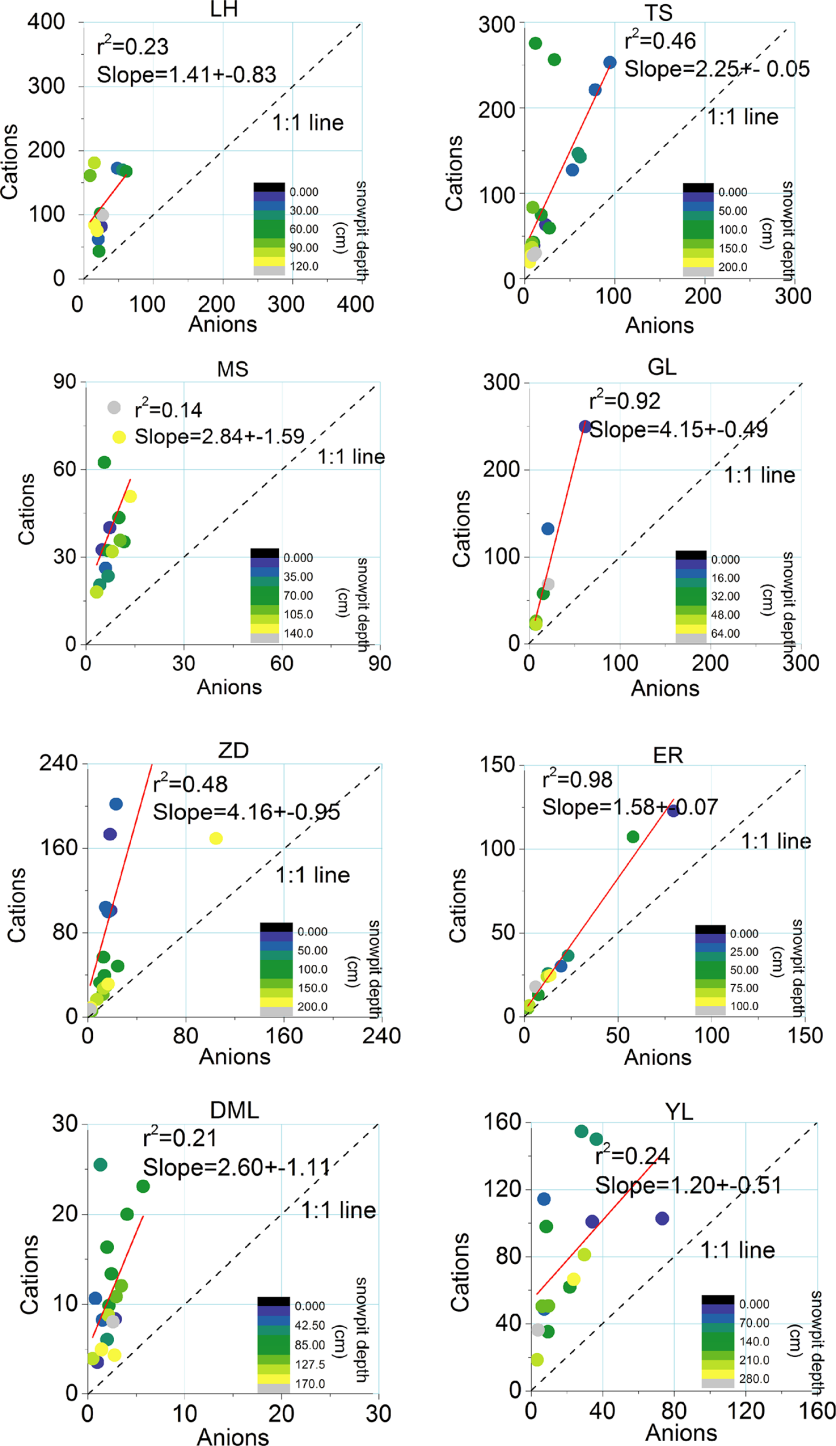
Charge balance (μeq/L) between cations [Na^+^ + K^+^ + Ca^2+^ + Mg^2+^ + NH_4_^+^] and anions [Cl^−^+ NO_3_^–^ + SO_4_^2–^] measured in TS, LH, MS, GL, ZD, ER, DML, and YL snow samples; colors indicate depth ranges. (TS, LH, MS, GL, ZD, ER, DML, and YL are the same as in Fig 2)

Measured ionic concentrations reveal a large anion charge deficit in the eight snowpits (Fig 4). Because carbonates are often high in mineral dust, which is widespread on the TP, the anion deficits are likely due to the lack of CO_3_^2–^ and HCO_3_^–^ [35], which is often enriched in mineral dust. The poor relationship between the sum of cations (Σ+) and anions (Σ−) from LH, MS, DML and YL maybe indicate the different postdepositional processes, especially the process if ionic elution, which not only resulted in enriching of some ions but also smoothed fluctuations of ionic concentrations [32]. As shown in LH snowpit, Ca^2+^ occurred a peak at the depth of 80–90 cm, while Na^+^ and SO_4_^2-^ low concentrations (Fig 3). On the other hand, input of local-regional mineral dust (microparticles) deposited on the glacier surface also affects the glaciochemical concentrations and charge balance [31]. For instances in YL snowpit (Fig 3), there existed obvious discrepancies between dust and ions variations. For GL and ER, the Σ+ was significantly correlated with the Σ− (Fig 4), suggesting the ions were undergoing the common postdepositional processes. Results of ER snowpit with anion deficit, relatively lower crustal ions (Ca^2+^ and Mg^2+^), and relatively high Na^+^, Cl^-^and SO_4_^2-^ were very similar to previous measurements on Himalayan samples, suggesting regional characteristics associated with the location’s distance from air pollution centers and high altitude [33, 35–36]. Besides, different environmental characteristics with different altitude and latitude, including the wind strength (affect dust deposition), the area of bare rock and rock weathering extent, may also have an important impact on ionic charge balance. ZD glacier with large area of bare rock surrounded, dust and ionic concentrations in snowpit showed high values (Table 2), comparable to that in the northern TP.

### 3.3 Spatial distributions of microparticles and major ions

**Fig 5:**
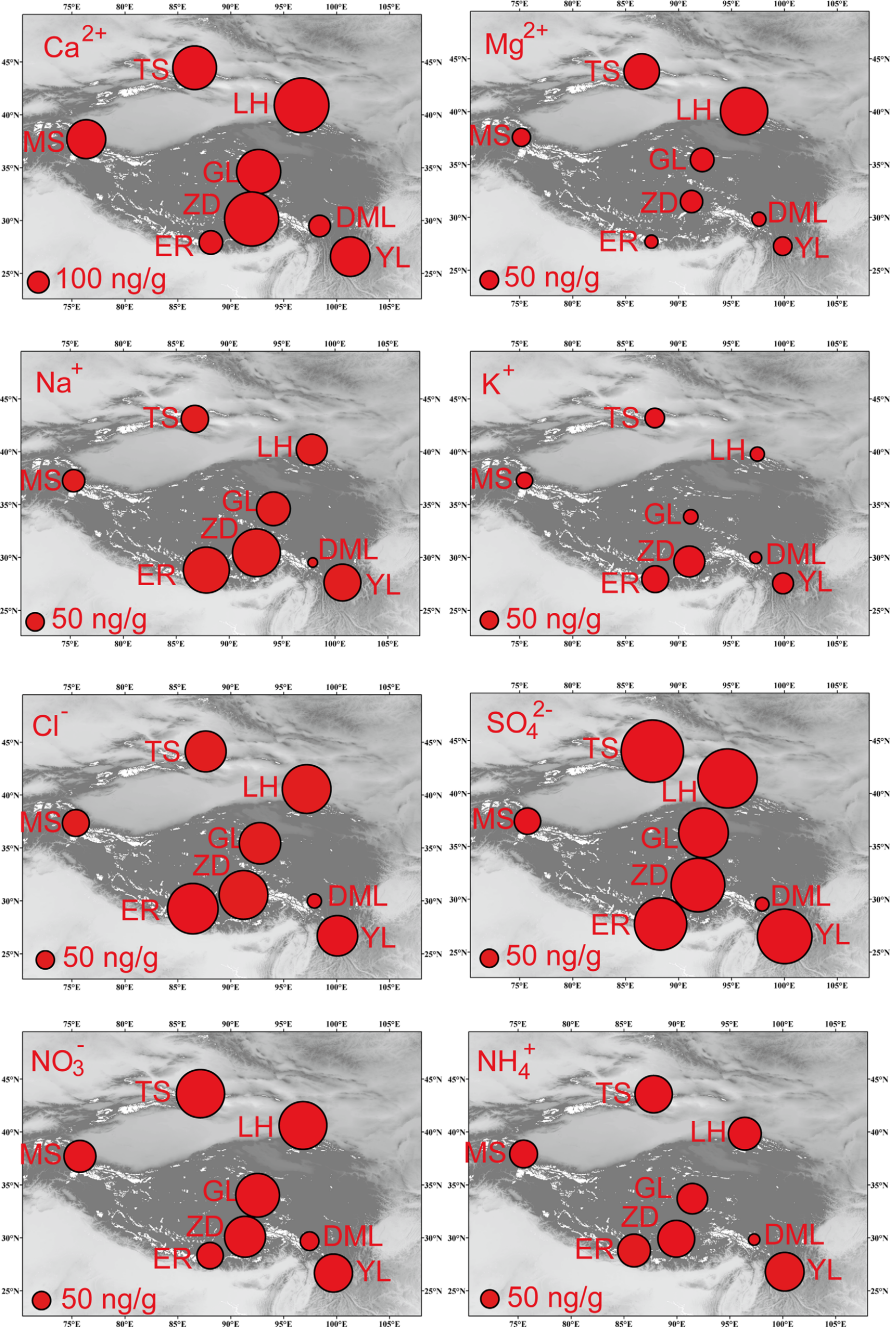
Spatial distributions of microparticles and major ions from the snowpits in the Tibetan Plateau. (TS, LH, MS, GL, ZD, ER, DML, and YL are the same as in Fig 2.)

As shown in Fig 5, higher concentrations of microparticles, Ca^2+^, and Mg^2+^, were generally occurred in TS, LH, and MS, over the desert areas in the northern and western areas of the TP (Taklimakan desert in the Tarim Basin). Concentrated Cl^–^, Na^+^, and K^+^ values were generally spread over the saline lake regions (e.g. Qiangtang plateau in inland TP) [22] and the southern TP (ER) profoundly affected by oceanic moisture from the Indian Ocean. Patterns of SO_4_^2–^, NO_3_^–^, and NH_4_^+^, which may be affected by anthropogenic emissions of SO_2_ and NO_x_, commonly showed higher values near cities, such as TS close to Ürümqi city, LH close to Lanzhou and Xining city, ZD near Lhasa city, and YL near Lijiang city. In the southern central-Himalayan regions (e.g., Khumb valley and Island peak), concentrations of microparticles and crustal ions (e.g., Ca^2+^) have much lower values compared to the northern sites, but, nitrite and sulfate are higher [37]. Previous studies have indicated that polluted aerosols (rich in SO_4_^2-^and NO_3_^-^) can spread across the southern area of the TP, which was defined by the towering peaks of Himalayas [4, 38]. When deposited on the glaciers, these aerosols may generate slightly higher SO_4_^2–^, NO_3_^–^, and NH_4_^+^ values, especially on the southern slopes of the Himalayas.

## 4. Potential Sources of Glaciochemistry

**Fig 6:**
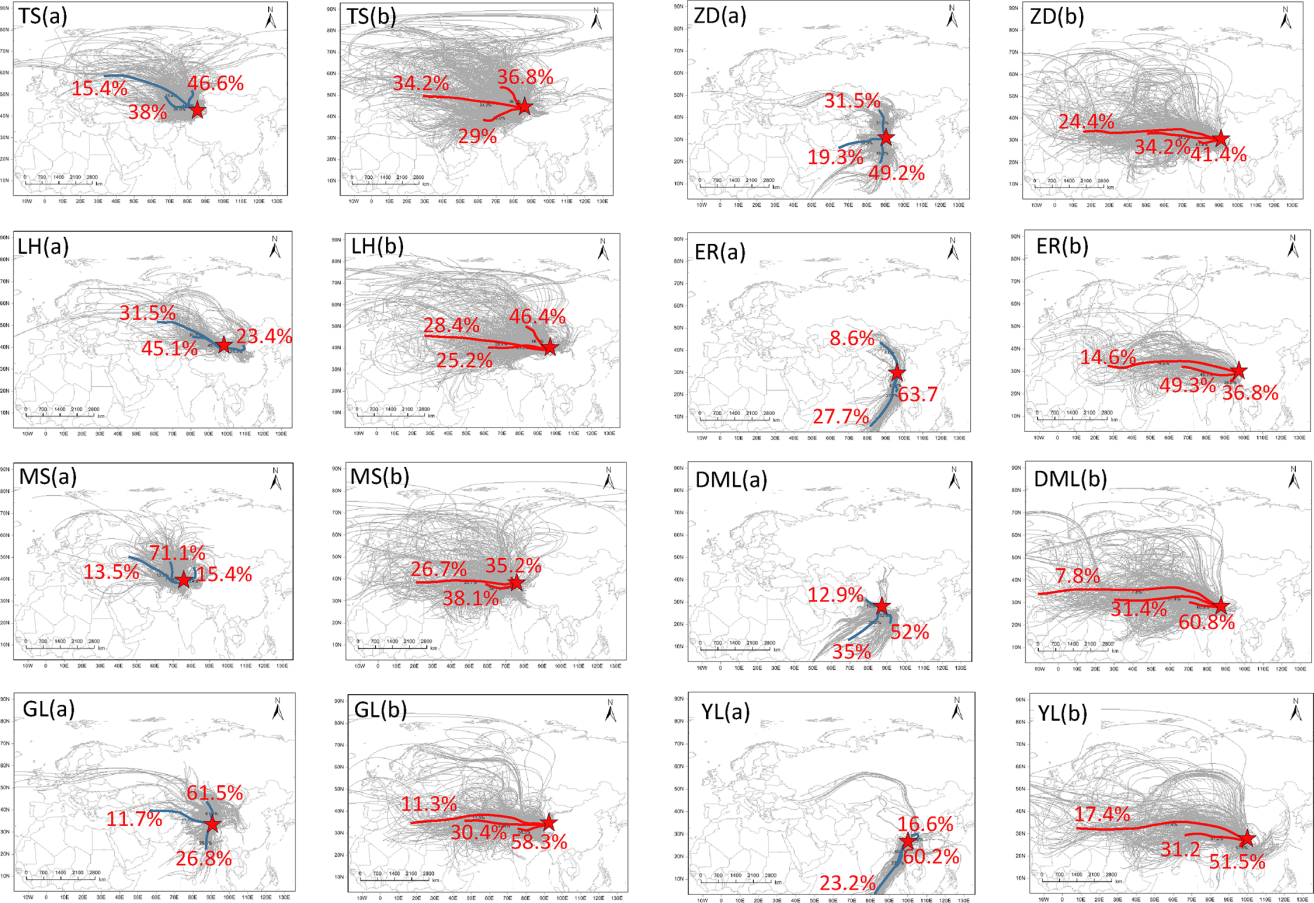
Air mass 7-day backward trajectories simulated by HYSPLIT model in the studied glaciers based on the seasonal divisions in Table 1: (a) monsoon season and (b) non-monsoon season. (TS, LH, MS, GL, ZD, ER, DML, and YL are the same as in Fig 2.)

To further investigate the inter-species relationships and common structure within the glaciochemical data, we performed principal component analysis (PCA) on major ions and microparticles records from the above eight snowpits. PCA is a multivariate technique that uses an orthogonal transformation to convert a set of observations of possibly related variables into a set of values of linearly uncorrelated variables. Its goal is to extract the important information from the table, to represent it as a set of new orthogonal variable, and to display the pattern of similarity of the observation and of the variables as points in maps [39]. This transformation has been defined in such way that the first principal component has the largest variance possible under the constraint that it is orthogonal to the preceding components. PCA results were summarized in Tables 3–10. In order to address the spatial differences of glaciochemistry in the snowpits and to consider the effects of the sources of moisture, we used the HYSPLIT model (details in S2 Text) to trace the air mass trajectories back to their source regions. Using seasonal divisions (see in Fig 2), air mass trajectories were calculated in the monsoon and non-monsoon seasons, respectively (Fig 6).

**Table 3:** Principal component analysis of microparticles and major ions in snowpit from Ürümqi Glacier No. 1, Tien Shan (TS).

TS	Component		
	PCA1	PCA2	PCA3
**Particle**	0.86	1.19	**92.0**
**Na**^**+**^	**90.1**	8.64	0.92
**NH**_**4**_^**+**^	8.94	**88.17**	0.83
**K**^**+**^	***24*.*4***	***17*.*64***	***17*.*3***
**Mg**^**2+**^	0.18	0.34	**94.5**
**Ca**^**2+**^	***37*.*7***	***20*.*34***	***32*.*8***
**Cl**^**–**^	**87.1**	10.56	0.94
**SO**_**4**_^**2-**^	***46*.*4***	***48*.*30***	0.13
**NO**_**3**_^**–**^	11.4	**84.27**	0.48
**% of Variance**	34.1	31.1	26.7
**Cumulative %**	34.1	65.2	91.9

**Table 4:** Principal component analysis of microparticles and major ions in snowpit from Laohugou Glacier No.12, Qilian Mountain (LH).

LH	Component		
	PCA1	PCA2	PCA3
**Particle**	23.3	**62.6**	8.29
**Na**^**+**^	20.07	0.0004	**75.9**
**NH**_**4**_^**+**^	**87.4**	2.69	0.48
**K**^**+**^	0.58	0.66	**95.1**
**Mg**^**2+**^	8.76	**89.5**	0.67
**Ca**^**2+**^	3.53	**91.6**	0.62
**Cl**^**–**^	***51*.*4***	4.71	***38*.*7***
**SO**_**4**_^**2–**^	**79.2**	3.96	5.62
**NO**_**3**_^**–**^	**89.3**	1.02	3.42
**% of Variance**	40.4	28.5	25.4
**Cumulative %**	40.4	68.9	94.3

**Table 5:** Principal component analysis of microparticles and major ions in snowpit from Muztagata Glacier (MS).

MS	Component		
	PCA1	PCA2	PCA3
**Particle**	0.27	0.45	**72.8**
**Na**^**+**^	**91.0**	0.05	0.45
**NH**_**4**_^**+**^	1.51	**76.56**	0.15
**K**^**+**^	**58.8**	13.10	0.10
**Mg**^**2+**^	0.85	1.56	**92.0**
**Ca**^**2+**^	0.01	11.76	**81.5**
**Cl**^**–**^	**96.4**	0.04	0.24
**SO**_**4**_^**2–**^	7.95	**62.73**	4.33
**NO**_**3**_^**–**^	0.18	**88.36**	0.96
**% of Variance**	28.6	28.3	28.1
**Cumulative %**	28.6	56.9	84.9

**Table 6:** Principal component analysis of microparticles and major ions in snowpit from Guoqu Glacier, Mt. Geladiandong (GL).

GL	Component		
	PCA1	PCA2	PCA3
**Particle**	***35*.*5***	**54.6**	2.28
**Na**^**+**^	**54.3**	***38*.*1***	6.92
**NH**_**4**_^**+**^	6.45	0.83	**90.8**
**K**^**+**^	22.3	**59.91**	15.3
**Mg**^**2+**^	**70.4**	17.1	12.4
**Ca**^**2+**^	**61.5**	26.8	5.06
**Cl**^**–**^	**62.9**	25.3	10.1
**SO**_**4**_^**2–**^	**63.8**	29.1	6.86
**NO**_**3**_^**–**^	**74.5**	13.8	11.6
**% of Variance**	50.2	29.5	17.9
**Cumulative %**	50.2	79.7	97.6

**Table 7:** Principal component analysis of microparticles and major ions in snowpit from Zhadang Galcier, Mt. Nyainqengtanglha (ZD).

ZD	Component		
	PCA1	PCA2	PCA3
**Particle**	8.38	**85.3**	3.39
**Na**^**+**^	**81.8**	14.4	0.76
**NH**_**4**_^**+**^	**79.4**	10.3	8.07
**K**^**+**^	**88.7**	6.40	0.20
**Mg**^**2+**^	10.6	**86.8**	1.61
**Ca**^**2+**^	10.3	**85.2**	0.18
**Cl**^**–**^	**94.7**	2.00	0.81
**SO**_**4**_^**2–**^	**76.2**	18.72	0.02
**NO**_**3**_^**–**^	4.51	**71.2**	22.3
**% of Variance**	50.5	42.2	4.15
**Cumulative %**	50.5	92.7	96.9

**Table 8:** Principal component analysis of microparticles and major ions in snowpit from East Rongbuk Glacier, Mt. Everest (ER).

ER	Component		
	PCA1	PCA2	PCA3
**Particle**	0.30	2.22	**97.4**
**Na**^**+**^	**63.5**	33.9	2.28
**NH**_**4**_^**+**^	**87.4**	10.3	0
**K**^**+**^	**82.6**	15.9	0.69
**Mg**^**2+**^	24.30	**71.1**	3.57
**Ca**^**2+**^	**82.3**	13.3	0.05
**Cl**^**–**^	**71.1**	26.1	2.43
**SO**_**4**_^**2–**^	**59.9**	37.6	2.28
**NO**_**3**_^**–**^	20.9	**76.2**	1.82
**% of Variance**	54.7	31.9	12.3
**Cumulative %**	54.7	86.6	98.9

**Table 9:** Principal component analysis of microparticles and major ions in snowpit from Demula Glacier (DML).

DML	Component		
	PCA1	PCA2	PCA3
**Particle**	**78.6**	11.3	2.72
**Na**^**+**^	1.30	**82.8**	1.06
**NH**_**4**_^**+**^	**24.9**	10.11	**24.9**
**K**^**+**^	6.25	**90.8**	0.26
**Mg**^**2+**^	**89.5**	6.15	0.02
**Ca**^**2+**^	**86.9**	3.69	1.35
**Cl**^**–**^	4.62	93.5	0.24
**SO**_**4**_^**2–**^	24.0	0.14	**47.9**
**NO**_**3**_^**–**^	2.72	0.10	**85.2**
**% of Variance**	35.4	33.2	18.2
**Cumulative %**	35.4	68.6	86.8

**Table 10:** Principal component analysis of microparticles and major ions in snowpit from Yulong Snow Mountain (YL).

YL	Component		
	PCA1	PCA2	PCA3
**Particle**	0.40	**96.0**	1.28
**Na**^**+**^	**89.5**	2.16	2.28
**NH**_**4**_^**+**^	**93.9**	1.02	0.01
**K**^**+**^	**79.9**	0.85	0.37
**Mg**^**2+**^	0.29	**50.7**	***38*.*7***
**Ca**^**2+**^	0.16	3.20	**93.1**
**Cl**^**–**^	**91.8**	0.32	0.48
**SO**_**4**_^**2–**^	**79.7**	0.0016	9.42
**NO**_**3**_^**–**^	**49.3**	4.54	10.8
**% of Variance**	53.9	17.6	17.4
**Cumulative %**	53.9	71.5	88.9

### 4.1 Northern Tibetan Plateau

In the northern TP area, the PCA results exhibited some common features (Table 3). At TS, the first component (PCA1 hereafter, 34.1% of total variance) consisted primarily of Na^+^ and Cl^–^, while the second component (PCA2 hereafter, 31.1% of total variance) consisted primarily of NH_4_^+^ and NO_3_^–^indicating anthropogenic source. PCA1 and PCA2 showed nearly equal concentrations of SO_4_^2–^. While Mg^2+^ and microparticles dominated in the third component (PCA3 hereafter), indicating that the main sources of major ions in TS was crustal dust from arid locations near the glacier.

At the LH snowpit (Table 4), PCA1 accounted for 40.4% of the total variance and was composed mainly of NO_3_^–^, NH_4_^+^, SO_4_^2–^, and Cl^–^, indicating large impact of the anthropogenic input. PCA2 accounted for 28.5% of the total variance and was composed of Ca^2+^, Mg^2+^, and microparticles, representing the crustal dust impact. K^+^ and Na^+^ were the main constituents of PCA3, indicating a different crustal source, possibly the weathering of local rocks and saline lakes.

In the MS region of the western TP (Table 5), PCA1 consisted primarily of Na^+^, K^+^, and Cl^–^, accounting for 28.6% of the total variability. PCA2 accounted for 28.3% of the total variability and consisted primarily of anthropogenic species (e.g., NO_3_^–^, NH_4_^+^, and SO_4_^2–^). Mg^2+^, Ca^2+^, and microparticles, representative of crustal dust deposition in the MS glacier, were presented in PCA3. PCAs results might refer to similar two types of crustal origins and anthropogenic sources as indicated above.

At TS, LH, and MS, most of the moisture comes from northern Eurasia, supplied from the northern Atlantic Ocean and the Arctic areas. During the monsoon season (Fig 6-TS(a), LH(a), MS(a)), the air masses travel generally from western Asia and/or northern Eurasia, and are subsequently influenced by re-evaporation from the surrounding regions and the Arctic air mass. During the non-monsoon seasons (Fig 6-TS(b), LH(b), MS(b)), the air masses originate mainly in western Asia, the northern Atlantic Ocean, and the Arctic.

Tian et al. [25] have found that, in the Tien Shan region, there is an apparent temperature effect on δ^18^O values due to differing seasonal evaporation conditions, with enriched values occurring in summer and depleted values occurring in winter. The air mass clearly originated in the east of the industrial regions (such as Lanzhou and Xining city) and may be the source of the anthropogenic pollution deposited on the glacier, as demonstrated by the concentrations of SO_4_^2–^, NO_3_^–^, and NH_4_^+^ in the same principal component (Table 3). Because of its location adjacent to Asian arid regions, the MS glacier provided a unique opportunity to study atmospheric dust deposition [13, 24, 40]. During the monsoon season, the trajectories were more localized, resulting in high concentrations of crustal components. During the non-monsoon seasons, air masses originated mainly in West Asia (Iran-Afghanistan Plateau) and Central Asia. The results leaded to the visible seasonality of glaciochemistry records with high monsoon values and low non-monsoon values, similar to the patterns at LH (Fig 3), suggesting higher dust loadings and stronger winds in the summer over the Pamir [41]. Besides, LH glacier is located near industrial regions (Hexi Corridor in western China) where SO_2_ and NO*x* emissions are high, suggesting that these species may be primarily due to anthropogenic pollution.

### 4.2 Inner Tibetan Plateau

In the inner TP, PCA of GL snowpit cannot be clearly distinguished in each component. Na^+^, K^+^, Ca^2+^, SO_4_^2–^, and microparticles were present in both PCA1 and PCA2 (Table 6). NH_4_^+^ was concentrated mainly in PCA3, suggesting a biogenic source (pasture or grass burning) in the TP [22]. The GL snowpit chemistry consists mainly of components from crustal and other sources, such as local minerals or ions from biogenic activity [16].

Result of the ZD showed that PCA1 (Table 7) consisted mainly of Cl^–^, K^+^, Na^+^, NH_4_^+^, and SO_4_^2–^ which indicated a mixture sources. PCA2 was dominated by Mg^2+^, Ca^2+^, and microparticles for a clear crustal input. Discrepancies between ZD and GL may be a result of lake salt impact (e.g., Nam Co lake with an area of 1920 km^2^) and human activity (e.g., pasture, combustion) contributions in the ZD region than that in the GL region.

In the inner TP, the air masses originated mainly from three directions: the Bay of Bengal, Thar Desert area, and Central Asia in the monsoon seasons. Warm and wet air mass mostly derived from the Indian Ocean as shown in Fig 6-GL(a) and ZD(a) would produce plentiful precipitation, resulting in the more negative δ^18^O and less ionic concentrations (Figs 2 and 3). However, air mass from the arid regions can bring massive crustal input affecting the PCA results as Na^+^, K^+^, Ca^2+^, SO_4_^2–^, and microparticles loaded in the same component (Table 3). While the air masses were mainly from arid and semi-arid areas (West Asia and western TP) during the non-monsoon periods as shown in Fig 6-GL(b) and ZD(b). The continental air masses resulted in the higher loading of major ions and dust, especially the crustal ions (e.g., Ca^2+^) (Fig 3), and even formed the visible dust layers (Fig 2). Our analysis is consistent with the results of others in the region [16, 42]. It is likely that high crustal values during the non-monsoon season are a product of the heavy dust storms in the arid regions of central and southern Asia [43].

### 4.3 Southern to southeastern Tibetan Plateau

South to southeast marginal region of the TP is largely influenced by the Indian monsoon, with moisture origins shifting between the Bay of Bengal and the southern Indian Ocean [26]. At the ER snowpit, PCA1 was largely dominated by Na^+^, K^+^, NH_4_^+^, Cl^–^, SO_4_^2–^, and Ca^2+^ (Table 8), accounting for 54.7% of the total variance with impact of marine aerosol and crustal dust. PCA2 had high concentrations of Mg^2+^ and NO_3_^–^ contributing 31.9% of the total variance. Microparticles dominated PCA3, which had a paucity of crustal ions.

PCA results clearly distinguished the sources of major ions and microparticles in the DML glacier. Ca^2+^, Mg^2+^, and microparticles dominated in PCA1 (Table 9), indicating a dust source. PCA2 was dominated by Na^+^, K^+^, and Cl^–^, indicating a marine or lacustrine sources. PCA3 had high NH_4_^+^, SO_4_^2–^, and NO_3_^–^, possibly indicating anthropogenic influences from long-distance /local areas, evidenced by low-modular-weight organic acids in the precipitation of the southeastern TP [44].

YL glacier is located near human settlements, 25 km north of Lijiang city, where anthropogenic activity and traffic emissions contribute to atmospheric pollution [45]. PCA results at YL showed that Na^+^, K^+^, NH_4_^+^, Cl^–^, SO_4_^2–^, and NO_3_^–^ represent 53.9% of the total variance in PCA1 (Table 10), indicating an anthropogenic influence (i.e., vehicle emission and biomass combustion) [36, 45]. PCA2 was dominated by microparticles and Mg^2+^, while PCA3 was dominated by Ca^2+^ and Mg^2+^, indicating the impact of dust deposition on the glacier.

Air masses generally originated from the Bay of Bengal and Thar Desert area with small influences from northerly air masses during the monsoon season (Fig 6-ER(a), DML(a) and YL(a)). Abundant precipitation leads to more negative δ^18^O values. During the non-monsoon season, air masses moved generally from Central/West Asia and Africa (Fig 6-ER(b), DML(b) and YL(b)), both of which have vast arid and semi-arid regions. These two air mass patterns may lead to different microparticle records in the snows of the monsoon or non-monsoon seasons.

### 4.4 Potential dust sources

**Fig 7:**
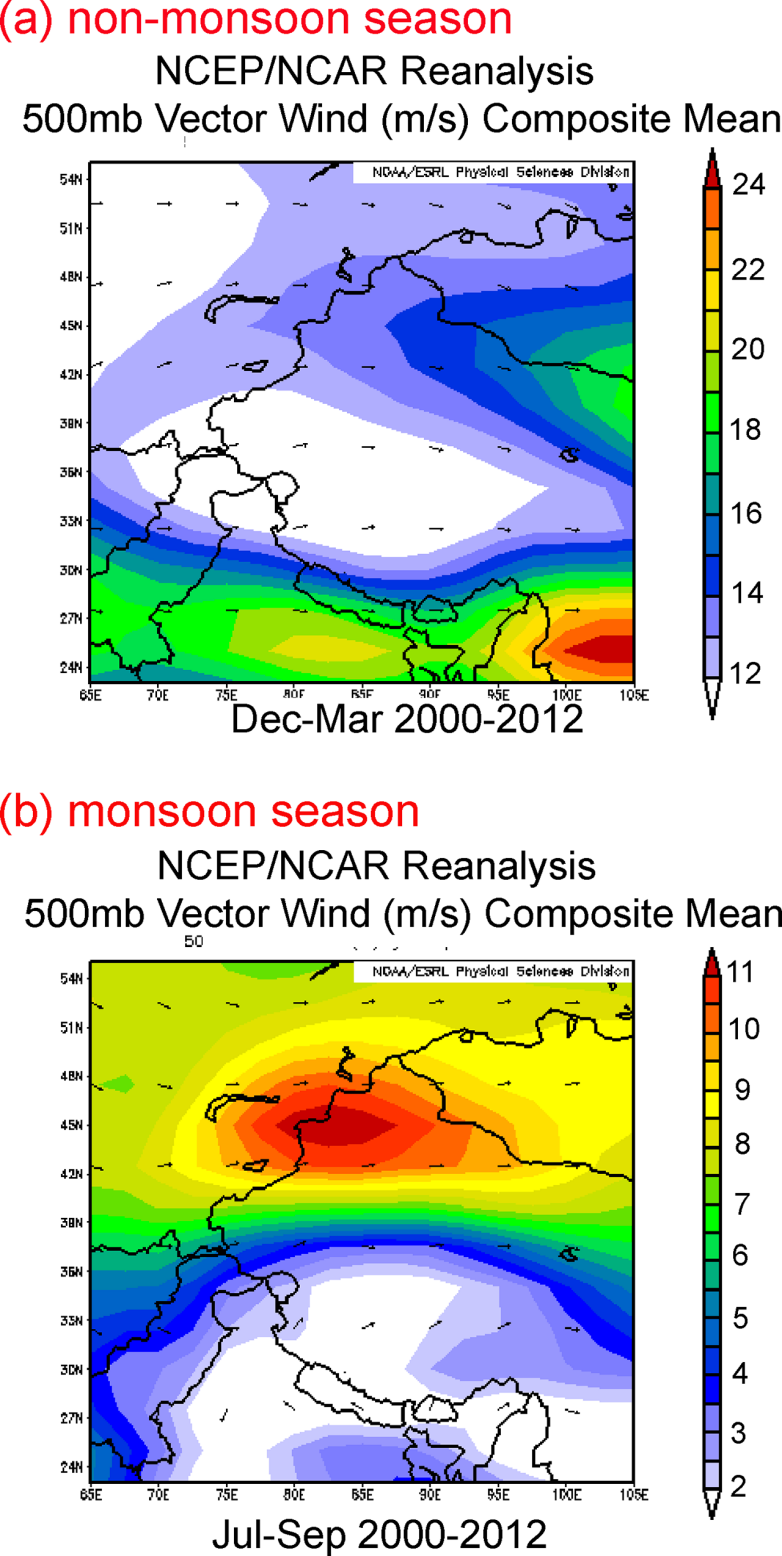
Vector winds of (a) non-monsoon season and (b) monsoon season based on the NCEP/NCAR reanalysis data in the studied regions.

Previous studies have found that frequent dust storms occurred in western China, mainly in spring [43]. Lying between the Tien Shan Mountains to the north and the Kunlun Mountains to the south, the Taklimakan Desert (Fig 1) in western China is one of the Earth’s largest shifting-sand deserts. The vertical distribution of CALIPSO backscatter indicates that non-spherically shaped dust aerosols were suspended above the dust source regions from near the ground to an altitude of approximately 9 km [46]. Moderate resolution imaging spectroradiometer (MODIS) images of the northern edge of the Tibetan Plateau revealed that a dust storm developed over the Taklimakan Desert on 30 January 2005 (S1 Fig), which may have affected dust deposition in the Kunlun and Qilian Mountains. The analysis of REE (Rare Earth Elements) also suggested that the Taklimakan desert might be the major source area for dust in Qilian Shan snow, promoted by perennial westerlies [40]. This suggests the possible long-range transport of entrained dust aerosols via upper tropospheric westerlies, which is then transported to the northern slope of TP [46–47]. Our results of HYSPLIT model suggested that TS glacier was impacted mainly by the westerlies and arctic air masses (Fig 6). REE result showed that dust in the TS regions mainly originated from the Taklimakan and Junggar deserts and from arid regions of mid- to long-range source areas in upwind Central Asia [13, 40]. The MS glacier is located in the east Pamir and perennially impacted d by westerlies [40]. The air masses reaching the MS regions come predominantly from west and Central Asia. However, the wide compositional range of Sm/Nd and strong negative Eu anomaly in the MS ice core also indicate a local contribution to glacial chemistry is a minor possibility [40]. In contrast, the arid and dry areas on the western TP may be the major source regions for atmospheric dust [43]. The vector winds based on NCEP/NCAR reanalysis data (Fig 7A) indicate that, during the non-monsoon seasons, the westerlies crossed desert regions and transported more dust on the TP glaciers (except DML), as demonstrated by the high microparticle input.

During the monsoon seasons, the central to southern TP is strongly influenced by the Indian monsoon (Fig 7B), which carries moisture and provides the most of precipitations on the TP. The haze observed from MODIS accumulates along the Himalaya Mountains, forming a strip of white-gray that obscures the ground (S2 Fig). By combining aerosol optical depth (AOD) data and backward trajectories, and verifying these with satellite data, Lüthi et al. [38] found that the atmospheric brown clouds from the South Asia were definitely able to reach the TP by crossing the Himalayas. AOD index over the TP indicated that the northern and western TP had higher aerosol loadings than the southern TP, suggesting a dust transport route mostly from the northwest to the southeast [13]. Even in summer, transport of dust aerosols originating from the Taklimakan desert was likely to be important factor determining inter-annual variation of AOD over the TP [48]. Thus, dust transported from the desert southward into the inner TP at an altitude of 4–7 km [49], was a likely constituent of the snow chemistry of glaciers in this region.

In some cases, local dust is a major contributor to TP glaciochemistry, a result of local topography or the complex wind systems in the mountain valleys. Zou et al. [50] found that the dominant down-valley flow in the Rongbuk valley is mostly attributable to thermally driven winds (valley, mountain, and glacier). Three-dimensional footprints, derived from the particle dispersion model for the Rongbuk Monastery observational site, also clearly show influences from Mount Everest and the southern part of the valley [51], with a vertical extension of influence was as high as 2–3 km. Without detailed tracer elements and their isotopes, and the meteorological records, the specific source of snow chemistry still exists uncertainties.

## 5. Conclusions

Investigations of glaciochemistry are critical for understanding environmental conditions. In this study, we analyzed the snow chemistry (δ^18^O, microparticles, Na^+^, K^+^, Mg^2+^, Ca^2+^, NH_4_^+^, Cl^–^, SO_4_^2–^, and NO_3_^–^) from eight snowpits in the TP and its surroundings, and discussed their potential sources. This work is expected to significantly improve the interpretation of the detailed information available in the massive database of glaciochemical records from the high altitude glaciers of the TP.

The mean values of δ^18^O in snowpits showed more negative at ER in the southern TP and less negative at LH in the northern TP and Tien Shan, due to precipitation amount, temperature or the large-scale atmospheric moisture (e.g., Indian monsoon, westerlies) impact. On average, the major ions in the eight snowpits were dominated by Ca^2+^ (~40%), with important contributions from SO_4_^2–^ and NO_3_^–^. Spatial distributions indicated that concentrations of Ca^2+^ and Mg^2+^, typical crustal constituents, gradually increased from south to north among the eight sampled glaciers, with the maximum concentrations occurring at LH, near the desert areas of Northwest China. Measured ionic concentrations reveal a large anion charge deficit in the eight snowpits, likely due to the lack of CO_3_^2–^ and HCO_3_^–^. Discrepancies of charge deficit among the snowpits may be caused by the postdepositional processes, mineral dust deposition or local topography (e.g., rock area around the glacier, rock weathering) where glacier located.

Combined with PCA, our results showed that the glacier snow chemical compositions in the TP were dominated by crustal aerosols as demonstrated by the high concentrations of ions occurring during the non-monsoon seasons. Other sources, such as anthropogenic pollution, played an important role on chemical variations of glaciers near the human activity centers (e.g., YL and TS). These PCA results may be related to the truth that the vast northern regions of the study area are arid because of the long distance from the coast and the rain shadow effect of the surrounding mountain ranges. The larger, low-elevation desert basins (such as Gobi, Taklimakan, and Gurbantunggut deserts) near the study area also contribute to abundant crustal aerosols.

Tracing the backward air mass trajectories by using the HYSPLIT model and wind field analysis, our results also indicated the probably moisture/dust sources influencing the δ^18^O oscillations and the snow chemistry seasonality. Dissimilarities of air masses arriving at the studied glaciers suggested that, in the northern TP, the air masses travel generally from western/western Asia and/or northern Eurasia during monsoon season, and mainly from western Asia/central, the northern Atlantic Ocean, and the Arctic during the non-monsoon seasons. The westerlies influencing the MS, LH, and TS snowpits exhibited more significant temperature effects, resulting in a high δ^18^O in summer seasons. Ions in snow at TS, LH, and MS showed relatively higher concentrations. In the inner TP, the air masses originated mainly from the Bay of Bengal, Thar Desert area, and Central Asia during monsoon season, and arid and semi-arid areas during the non-monsoon periods. In the southern southeastern TP, air masses generally originated from the Bay of Bengal and Thar Desert area with small influences from northerly air masses during the monsoon season, and generally from Central/West Asia and Africa. The monsoon-related seasonality in the ER, YL, and ZD snowpits were attributable to moisture originating between the Bay of Bengal and the southern Indian Ocean, with a decreasing δ^18^O value during the monsoon season. This study will improve current understanding of the deposition and transport of glacial chemicals in the TP and surrounding areas.

## Supporting Information

S1 Fig Dust storm of Taklimakan Desert images from MODIS. Images from the Moderate Resolution Imaging Spectroradiometer (MODIS) on NASA’s Aqua satellite showing the desert with dust on 30 January 2005 (top) and a clear day on 2 November 2002 (bottom).On 30 January 2005, dust filled the bowl of the Taklimakan Desert in western China. The low-lying basin is ringed by towering mountains to the north and south; these mountains steal almost all the precipitation passing through the region, leaving the Taklimakan bone dry. (http://earthobservatory.nasa.gov/NaturalHazards/view.php?id=14583&eocn=related_to&eoci=related_image)
(TIFF)

S2 Fig Mixture of haze and dust in the Indo-Gangetic Plain. What may be a mixture of haze and dust is spread out in a band at the foothills of the Himalaya Mountains in northern India (occupying most of the scene) and Pakistan (at upper left) and in a second swath in the center of the scene. The haze stretches over the Mouths of the Ganges River (right center edge) and the Bay of Bengal to the south, forming a strip of white-gray that obscures the ground. Beyond the high peaks of the Himalaya, skies are clear over the Tibetan Plateau. This image was captured by the Moderate Resolution Imaging Spectroradiometer (MODIS) on NASA’s Terra satellite on 15 November 2004. (http://www.nasa.gov/topics/earth/features/terrain-heat-pump.html)
(TIFF)

S1 Table Sampling information for the studied glaciers in the Tibetan Plateau and adjacent areas. (PDF)

S1 Text Detailed information of sampling sites. (PDF)

S2 Text Introduction of HYSPLIT model. (PDF)
