# Supplementary figures and images for: Chemical Records in Snowpits from High Altitude Glaciers in the Tibetan Plateau and Its Surroundings
Source: PLoS One. 2016 May 17;11(5):e0155232. doi: 10.1371/journal.pone.0155232 (PMC4871367; doi:10.1371/journal.pone.0155232)

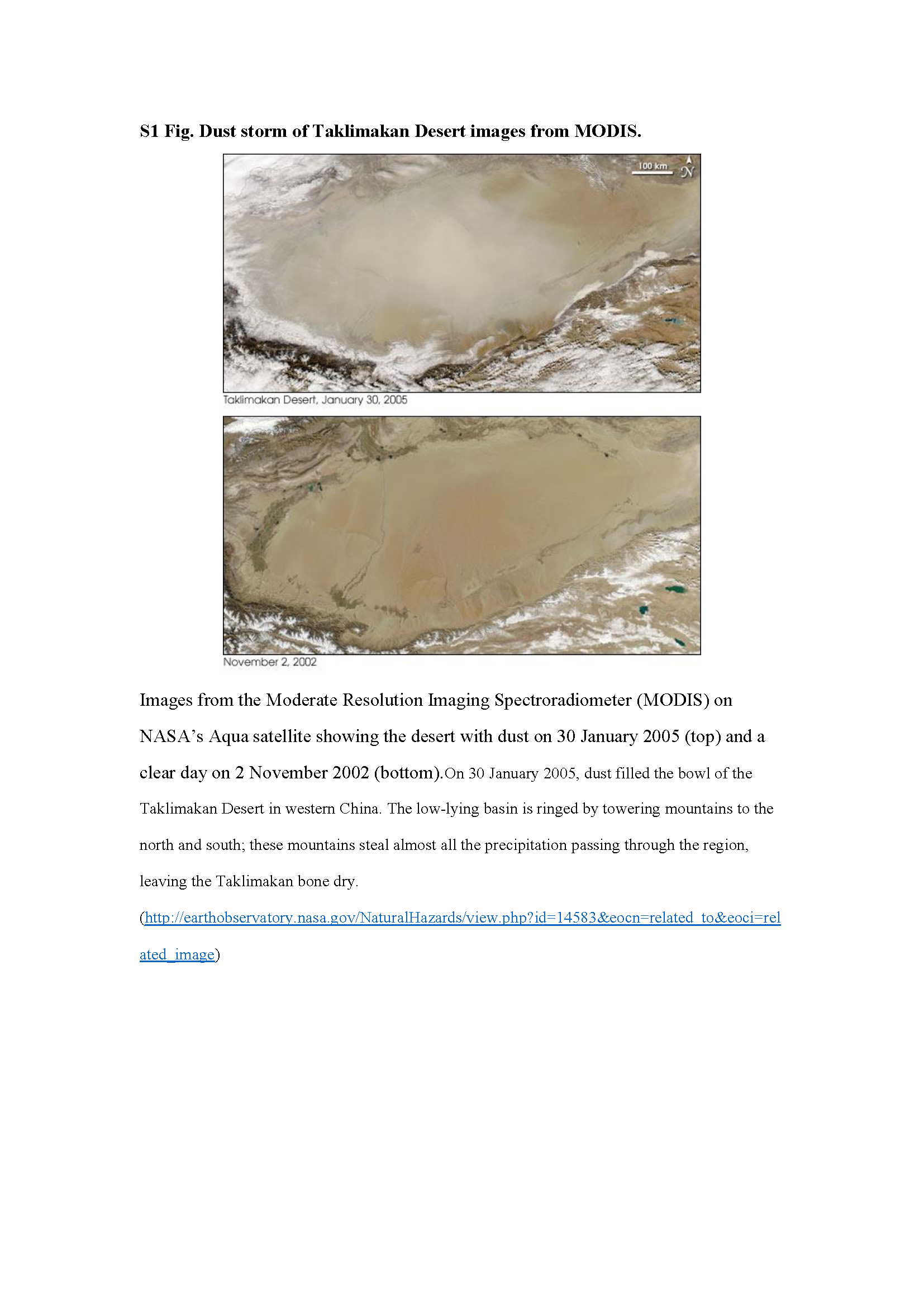

Supplement: S1 Fig — Images from the Moderate Resolution Imaging Spectroradiometer (MODIS) on NASA’s Aqua satellite showing the desert with dust on 30 January 2005 (top) and a clear day on 2 November 2002 (bottom).On 30 January 2005, dust filled the bowl of the Taklimakan Desert in western China. The low-lying basin is ringed by towering mountains to the north and south; these mountains steal almost all the precipitation passing through the region, leaving the Taklimakan bone dry. (http://earthobservatory.nasa.gov/NaturalHazards/view.php?id=14583&eocn=related_to&eoci=related_image) (TIFF) [file pone.0155232.s001.tiff]

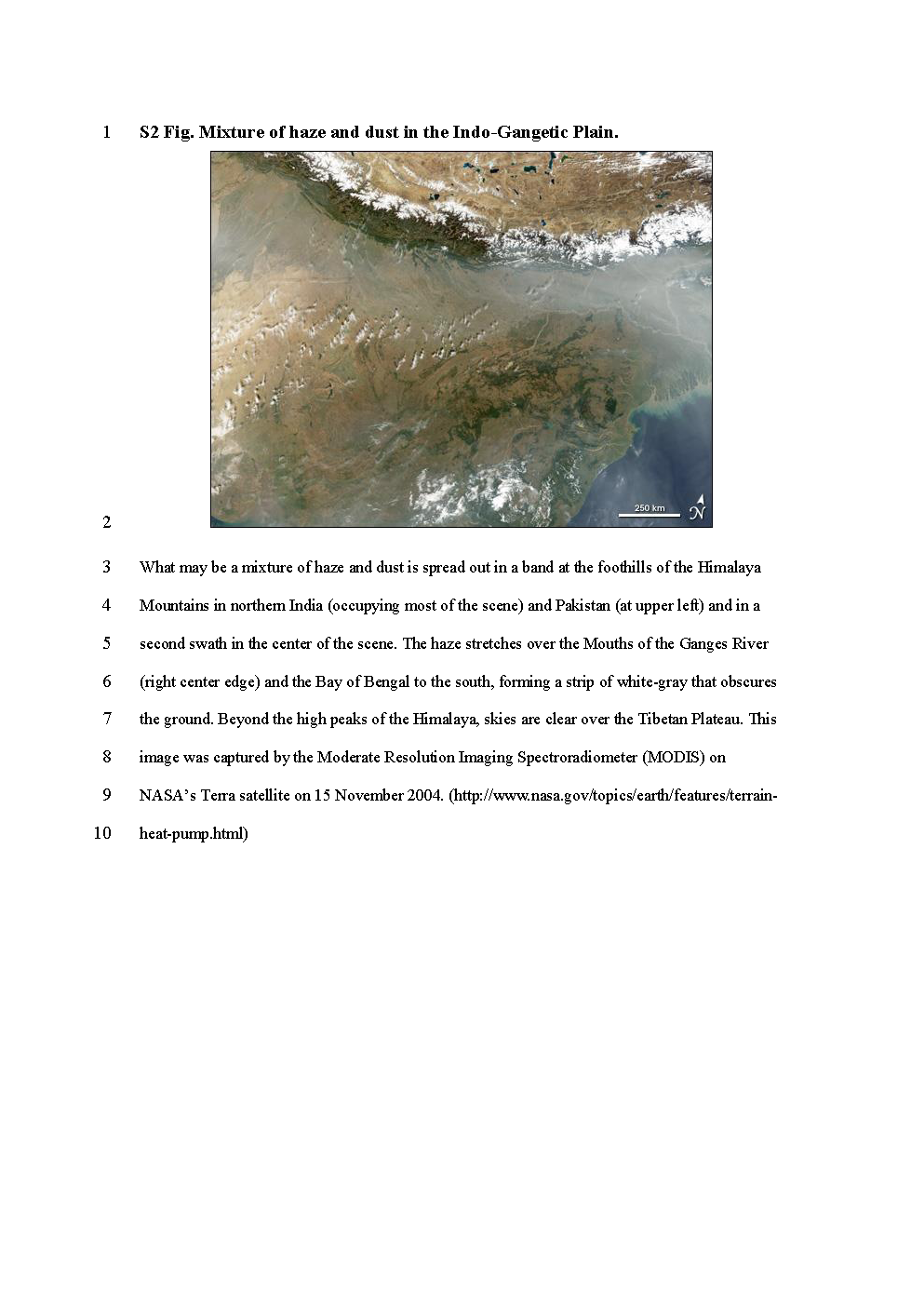

Supplement: S2 Fig — What may be a mixture of haze and dust is spread out in a band at the foothills of the Himalaya Mountains in northern India (occupying most of the scene) and Pakistan (at upper left) and in a second swath in the center of the scene. The haze stretches over the Mouths of the Ganges River (right center edge) and the Bay of Bengal to the south, forming a strip of white-gray that obscures the ground. Beyond the high peaks of the Himalaya, skies are clear over the Tibetan Plateau. This image was captured by the Moderate Resolution Imaging Spectroradiometer (MODIS) on NASA’s Terra satellite on 15 November 2004. (http://www.nasa.gov/topics/earth/features/terrain-heat-pump.html) (TIFF) [file pone.0155232.s002.tiff]
