# Supplementary material for: Chemical Records in Snowpits from High Altitude Glaciers in the Tibetan Plateau and Its Surroundings
Source: PLoS One. 2016 May 17;11(5):e0155232. doi: 10.1371/journal.pone.0155232 (PMC4871367; doi:10.1371/journal.pone.0155232)
Supplement: S1 Table — (PDF) [file pone.0155232.s003.pdf]

S1 Table. Sampling information for the studied glaciers in the Tibetan Plateau and adjacent areas.

Sites	Sampling Time	Latitude (°)	Longitude (°)	Elevation (m a.s.l.)	Snowpit depth (cm)	Sample numbers
**Ürümqi Glacier No. 1 (TS)**	2008-10-20	43.11	86.81	4063	215	22
**Laohugou Glacier No.12 (LH)**	2008-10-16	39.43	96.56	5026	125	13
**Muztagata Glacier (MS)**	2010-7-18	38.29	75.05	5725	145	15
**Guoqu Glacier (GL)**	2009-4-23	33.58	91.18	5765	65	7
**Zhadang Glacier (ZD)**	2009-5-7	30.47	90.65	5797	205	21
**East Rongbuk Glacier (ER)**	2009-5-18	28.02	86.96	6525	105	11
**Demula Glacier (DML)**	2008-9-21	29.36	97.02	5404	175	18
**Yulong Snow mountain (YL)**	2009-5-20	27.11	100.20	4747	282.5	15
