# Supplementary material for: Chemical Records in Snowpits from High Altitude Glaciers in the Tibetan Plateau and Its Surroundings
Source: PLoS One. 2016 May 17;11(5):e0155232. doi: 10.1371/journal.pone.0155232 (PMC4871367; doi:10.1371/journal.pone.0155232)
Supplement: S1 Text — (PDF) [file pone.0155232.s004.pdf]

S1 Text. Detailed information of sampling sites

Ürümqi glacier No. 1, Eastern Tienshan (TS)

On 20 October 2008, we collected cores from a snow pit at Ürümqi glacier No. 1, Tienshan Range (43.11°N, 86.81°E, 4063 m a.s.l) (S1 Table). Glacier No.1 is located at the headwaters of the Ürümqi River in the eastern Tien Shan Mountains of central Asia, only 120 km from Ürümqi city. The glacier is surrounded by vast deserts: the Gobi desert to the east, the Taklimakan desert in the Tarim basin to the south, the Peski Muyunkum and Peski Sary-Ishikotrau deserts to the west, and the Gurbantunggut desert in the Junggar basin to the north [1]. The mountain environment in this region includes a forest zone at 1500–2900 m a.s.l; alpine meadows above 2900 m a.s.l; and bare rock, glacial deposits, and permafrost above 3000 m a.s.l [2]. The region is dominated by a typical continental climate; the westerly jet prevails across these high mountains. Local valley winds dominate from March through September near the ground surface [3].

Laohugou glacier No.12, Qilian Mountain (LH)

Mt. Qilian, located at the northeast rim of the TP and near the Hexi corridor, has developed many glaciers. Since the area closely adjoins the inland arid and semi-arid territories, the study of the regional climate and environment from the glacier records is important. Laohugou Glacier No.12 (5Y448D0012) is located on the north slope of western Mt. Qilian and covers an area of 21.9 km^2 with a large accumulation zone [4]. The terminal elevation is 4260 m a.s.l. The slope of the glacier above 4500 m a.s.l is flat, while below 4500 m a.s.l there is an existing grand ice tower-forest and ice-river. We collected snow pit data at the No.12 Glacier in the accumulation zone to a depth of 135 cm (39.43°N, 96.56°E, 5026 m a.s.l) on 16 October 2008 (S1 Table).

Muztagata glacier (MS), the eastern Pamir

The Muztagata glacier on the western slope of Mt. Muztagata is located in the eastern Pamir mountain range and the western Tibetan Plateau, adjacent to the largest dust source regions in central Asia. According to the Glacier Inventory of China, 101 glaciers in the Mt. Muztagata region cover an area of 345 km^2 [5], making this one of the largest glacial areas in China [6]. The westerlies and the South Indian monsoon are the dominant atmospheric circulations over the Muztagata region [7]. On 18 July 2010, we collected a core from a 150 cm deep snow pit on the MS glacier (38.29°N, 75.05° E, 5725 m a.s.l) (S1 Table).

Guoqu glacier, Mt. Geladaindong (GL)

Mt. Geladaindong, the peak of the Tanglha Range and the source region of the Yangtze River, is located in the central TP. It represents the northernmost extent of the summer Indian monsoon [8]. Summer precipitation here is derived mainly from moisture transported by the Indian monsoon and regional convection, with limited precipitation during the winter due to the occasional influx of westerly disturbances.

On 23 April 2009, we obtained snow pit samples at the col of Guoqu Glacier (33.58°N, 91.18°E, 5765 m a.s.l) (S1 Table).

Zhadang glacier, Mt. Nyainqêngtanglha (ZD)

Zhadang Glacier (5Z225D0017) is located in the Nam Co basin, on the eastern saddle of Mt. Nyainqêntanglha, about 50 km southwest of the Nam Co Station for Multisphere Observation and Research (established by the Institute of Tibetan Plateau Research, CAS) [9]. During summer, most residents migrate from the northern to the southern bank of Nam Co basin for pasture [10]. Also, there are thousands of people driving to Nam Co basin for tourism. ZD Glacier mass balance, snow/ice records, and glacial-hydrology have been recorded since August 2005 and the results published by [9,11]. The Zhadang Glacier is at the boundary of the Indian monsoon and the continental climate of central Asia, offering a unique opportunity to describe and understand climatic changes and atmospheric chemistry over the TP [9]. We collected snow samples at the col of the glacier (30.47°N, 90.67°E, 5800 m a.s.l) on 7 May 2009 (S1 Table).

East Rongbuk glacier (ER), Mt. Everest

The East Rongbuk Glacier is in the Himalayan Range, surrounded by mountains ranging from 600 m to over 8000 m a.s.l., including Mt. Everest (8844 m a.s.l.). This location is near the boundary of the Indian monsoon-dominated wet and warm climate to the south and the westerly jet stream-dominated continental climate to the north. Southeast and southwest winds dominate the area as demonstrated by the mean wind field (based on the National Centers for Environmental Prediction/National Center for Atmospheric Research reanalysis data, NCEP/NCAR) at the geopotential height level of 500 hPa [12]. Snowfall at the glacier is caused by moisture transported by the Indian monsoon, local summer moisture coming from short-distance convective air masses (July to September), and by precipitation in other seasons associated with the westerly jet stream [13-14]. We collected samples on 18 May 2009 at a sampling site

located at Col of the East Rongbuk Glacier (28.02°N, 86.96°E, 6525m a.s.l.) (S1 Table).

Demula Glacier (DML)

Monsoonal temperate glaciers are occur in southeastern TP, near the Hengduan and Daxue mountains, the eastern Himalayas, and the central and eastern segments of the Mt. Nyainqêngtanglha range [6]. The region is characterized by high annual precipitation (1000~3000 mm) in the glacier-covered area, a much lower snowline (4200~5200 m a.s.l) than that of the continental glaciers in the western TP, and relatively high temperatures (mean annual temperature of –6°C). The monsoonal temperate glaciers in China cover an area of 13,200 km^2, accounting for 22% of the total glacier area in China [6]. These glaciers have been subject to intense ablation and accelerated retreat caused by global warming over the past century [15]. Below an elevation of 2700 m a.s.l., they are controlled by a sub-tropic climate; between 2700~4200 m a.s.l. they are dominated by a warm and semi-humid plateau climate; and above 4200 m a.s.l. they are dominated by a cold-humid plateau climate. Demula Glacier, located at the southeast rim of the TP, has a climate mainly controlled by the Indian monsoon. We collected the samples on 21 September 2008 on the accumulation zone of the glacier (29.36°N, 97.02°E, 5404 m a.s.l.) (S1 Table).

Yulong Snow Mountain (YL)

Yulong Snow Mountain, located in the southeastern part of the TP (only 15 km away from the Lijiang city), is the southernmost glacierized region in China, with typical temperate monsoonal glaciers that are characterized by both ablation and accumulation in summer[16-17]. Among these temperate glaciers, Baishui No.1 glacier is the largest, with an area of 1.52 km^2 and length of 2.7 km. Famous for its subtropical snow mountain spectacles (high mountains, deep valleys, meadows, shrubs, forest, and glaciers), Baishui No.1 glacier opened as a national glacier park in 1997. On 20 May 2009, we collected samples from a 295 cm deep snowpit at Baishui

No.1 glacier (27.11° N, 100.20°E, 4747 m a.s.l) (S1 Table). The regional climate is influenced mainly by the Asian summer monsoon, with annual average precipitation of 1000~3000 mm. The annual mean temperature at the ELA (equilibrium line altitude) is about –6°C. Summer (June-August) temperatures vary between 1°C and 5°C, while the temperatures of the ice ranges between 0°C and –4°C, but is usually above –1°C. Summaries of the sampling sites are listed in S1 Table.

References:

1. Li Z, Edwards R, Mosley-Thompson E, Wang F, Dong Z, You X, et al. Seasonal variability of ionic concentrations in surface snow and elution processes in snow-firn packs at the PGP1 site in Ürümqi glacier No.1, eastern Tien Shan, China. Ann Glaciol. 2006; 43: 250-256.
2. Luo, H., 1983. Hydrochemical features of the Glacier No. 1 in the source region of Urumqi River, Tianshan. J Glaciol Geocryol. 2008; 5(2): 55-64.
3. Zhang Y, Kang E, Liu C. The climatic features of Tianshen Urumqi River valley. J Glaciol Geocryol. 1994; 16(4): 333-341.
4. Wang Z, Liu C, You G. Glacier Inventory of China, I Qilian Mountains. Lanzhou: Lanzhou Institute of Glaciology and Cryopedology, Chinese Academy of Sciences. 1981. pp.1-249.
5. Liu C, Wang Z, Ding L. Glacier Inventory of China, IV Pamirs (drainage basins of Kaxgar River and others). Lanzhou: Gansu Culture Publishing House. 2001. pp. 23-52.
6. Shi Y. Concise Glacier Inventory of China. Shanghai Popular Science Press, Shanghai.
7. Wu G, Yao T, Xu B, Li Z, Tian L, Duan K, Wen L. Grain size record of microparticles in the Muztagata ice core. Sci China- Ser D. 2006; 49 (1): 10-17. http://dx.doi.org/10.1007/s11430-004-5093-5
8. Tian L, Masson-Delmotte V, Stievenard M, Yao T, Jouzel J. Tibetan Plateau summer monsoon northward extent revealed by measurements of water stable isotopes. J Geophys Res. 2001; 106(D22): 28081-28088.

9. Kang S, Huang J, Xu Y. Changes in ionic concentrations and $\delta 18O$ in the snowpack of Zhadang glacier, Nyainqentanglha mountain, southern Tibetan Plateau. Ann Glaciol. 2008; 49(1): 127-134.

10. Li C, Kang S, Zhang Q, Kaspari S. major ionic composition of precipitation in the Nam Co region, Central Tibetan Plateau. Atmos Res. 2007; 85: 351-360.

11. Gao T, Kang S, Krause P, Cuo L, Nepal S. A test of J2000 model in a glacierized catchment in the central Tibetan Plateau. Environ Earth Sci. 2012; 65(6): 1651-1659.

12. Ming J, Zhang D, Kang S, Tian W. Aerosol and fresh snow chemistry in the East Rongbuk Glacier on the northern slope of Mt. Qomolangma (Everest). J Geophys Res-Atmos. 2007; 112: D15307. http://dx.doi.org/10.1029/2007JD008618

13. Bryson R. Airstream climatology of Asia, in: Xu, Y. (Ed.), Proceedings of the International Symposium on the Qinghai-Xizang Plateau and Mountain Meteorology. Boston: American Meteorological Society. 1986. pp. 604–619. http://dx.doi.org/10.1007/978-1-935704-19-5_36

14. Yanai M, Wu G. Effects of the Tibetan plateau, in: Wang, B. (Ed.), The Asian Monsoon. Springer. 2006. pp. 513-549. http://dx.doi.org/10.1007/3-540-37722-0_13

15. He Y, Zhang Z, Theakstone WH, Chen T, Yao T, Pang H. Changing features of the climate and glacier in China’s monsoonal temperate glacier region. J Geophys Res. 2003; 108(D17). http://dx.doi.org/10.1029/2002JD003365

16. Li Z, He Y, Pu T, Jia W, He X, Pang H, et al. Changes of climate, glaciers and runoff in China's monsoonal temperate glacier region during the last several decades. Quat Intern.2010; 218(1-2): 13-28.

17. Wang S, He Y, Song X. Impacts of climate warming on alpine glacier tourism and adaptive measures: A case study of Baishui glacier No. 1 in Yulong Snow Mountain, southwestern China. J Earth Sci. 2010; 21: 166-178.
