# Supplementary material for: Chemical Records in Snowpits from High Altitude Glaciers in the Tibetan Plateau and Its Surroundings
Source: PLoS One. 2016 May 17;11(5):e0155232. doi: 10.1371/journal.pone.0155232 (PMC4871367; doi:10.1371/journal.pone.0155232)
Supplement: S2 Text — (PDF) [file pone.0155232.s005.pdf]

S2 Text. Introduction of HYSPLIT model

The Hybrid Single-Particle Lagrangian Integrated Trajectory (HYSPLIT) model (Version 4) is a complete system for computing a simple trajectory to complex dispersion and deposition simulations using either puff or particle approaches [1]. We chose the HYSPLIT model to obtain backward trajectory due to its compatibility with desktop computers and use of high-resolution meteorological data files. The meteorological data used for the computation of the trajectories came from the NCEP/NCAR reanalysis data archive maintained by NOAA Air Resources Laboratory (available online at http://www.arl.noaa.gov /ss/transport/ archives.html). The global estimate of the average lifetime of sulfate aerosol is about five days [2]; the aerosol lifetime over the INDOEX (Indian Ocean Experiment) field is estimated to be seven to eight days [3]. We compromised and used a method with a 7-day-long backward trajectory and a daily resolution to simulate the changing routes of air masses arriving at the sampling sites, ending at 12:00 (04:00 UTC) noon Beijing time each day. We calculated the backward trajectories at different levels because the study sites are located at different elevations. Because a trajectory is an indication of the general airflow rather than the exact pathway of an air mass, the analysis of a large number of trajectories in a climatological sense reduces the effects of individual errors [4]. Hence, a reasonable representation of the airflow to the studied sites was possible. The results of backward trajectory analysis are presented during monsoon seasons and non-monsoon seasons for the eight sites.

References:

1. Roland R, Hess G. Description of the HYSPLIT 4 Modeling System. NOAA Technical Memorandum ERL. 2004.
2. Langner J, Rodhe H. A global three dimensional model of tropospheric sulphate aerosol. J Atmos Chem. 1991; 13: 225-263.
3. Rasch PJ, Collins WD, Eaton BE. Understanding the Indian Ocean Experiment (INDOEX) aerosol distriutions with an aerosol assimilation. J Geophys Res. 2001; 106(D7): 7337-7355.
4. Harris JM, Kahl JD. A descriptive atmospheric transport climatology for the Mauna Loa observatory, using clustered trajectories. J Geophys Res. 1990; 95(D9): 13651–67.
